# Multiple independent losses of sporulation and peptidoglycan in the *Mycoplasmatales* and related orders of the class *Bacilli*

**DOI:** 10.1099/mgen.0.001176

**Published:** 2024-01-08

**Authors:** Christian J. Field, Kate L. Bowerman, Philip Hugenholtz

**Affiliations:** ^1^​ School of Chemistry and Molecular Biosciences, The Australian Centre for Ecogenomics, The University of Queensland, St Lucia, QLD 4072, Australia

**Keywords:** evolution, spore formation, cell envelope

## Abstract

Many peptidoglycan-deficient bacteria such as the *Mycoplasmatales* are known host-associated lineages, lacking the environmental resistance mechanisms and metabolic capabilities necessary for a free-living lifestyle. Several peptidoglycan-deficient and non-sporulating orders of interest are thought to be descended from Gram-positive sporulating *Bacilli* through reductive evolution. Here we annotate 2650 genomes belonging to the class *Bacilli*, according to the Genome Taxonomy Database, to predict the peptidoglycan and sporulation phenotypes of three novel orders, *RFN20*, *RF39* and *ML615J-28*, known only through environmental sequence surveys. These lineages are interspersed between peptidoglycan-deficient non-sporulating orders including the *Mycoplasmatales* and *Acholeplasmatales*, and more typical Gram-positive orders such as the *Erysipelotrichales* and *Staphylococcales*. We use the extant genotypes to perform ancestral state reconstructions. The novel orders are predicted to have small genomes with minimal metabolic capabilities and to comprise a mix of peptidoglycan-deficient and/or non-sporulating species. In contrast to expectations based on cultured representatives, the order *Erysipelotrichales* lacks many of the genes involved in peptidoglycan and endospore formation. The reconstructed evolutionary history of these traits suggests multiple independent whole-genome reductions and loss of phenotype via intermediate transition states that continue into the present. We suggest that the evolutionary history of the reduced-genome lineages within the class *Bacilli* is one driven by multiple independent transitions to host-associated lifestyles, with the degree of reduction in environmental resistance and metabolic capabilities correlated with degree of host association.

## Abbreviations

GTDB, Genome Taxonomy Database; KO, KEGG orthologue; KO, KEGG Orthology; LBA, long branch attraction; PBPs, penicillin-binding proteins.

## Impact Statement

Numerous major bacterial lineages lacking cultured representatives are being discovered through genome-centred metagenomics. These include the orders *RFN20*, *RF39* and *ML615J-28*, which are related to the order *Mycoplasmatales* in the phylum *Bacillota*. Here we show that two defining features of these lineages, loss of peptidoglycan from the cell envelope and loss of spore formation, have occurred independently on multiple occasions, indicating convergent reductive evolution probably as a consequence of transitioning to host-associated lifestyles.

## Data Summary

The authors confirm all supporting data, code and protocols have been provided within the article or through supplementary data files.

## Introduction

**Fig. 1.**
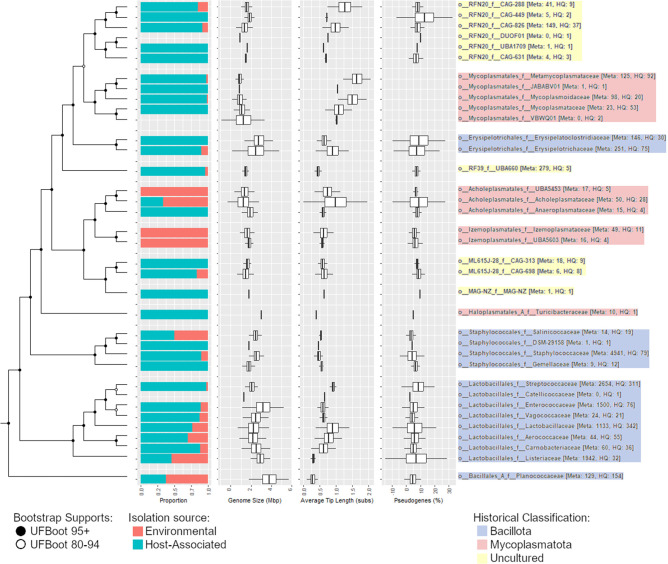
*De novo* maximum likelihood cladogram of the class *Bacilli*, trimmed to the ingroup families, showing the proportion of environmental and host-associated members, genome size, inferred evolutionary rate and percentage of pseudogenes. All statistics were averaged at the family level and boxplots show mean±3 sd. Pseudogenes were omitted for genomes using codon table 4. Interior node support is shown in the inset key. Colour highlighting indicates historical classifications and cultivation status of lineages of interest. Taxon names are prefixed with rank designations: o__, order; f__, family. The number of genomes used to infer ecological distribution for each family (Meta) and the number of high quality (HQ) genomes used for metabolic inference are shown in square brackets after the taxon names.

The phylum *Mycoplasmatota* (formerly known as the *Tenericutes*) encompasses the classes *Mollicutes* and *Candidatus* Izemoplasma, containing orders such as *Mycoplasmatales, Acholeplasmatales* and *Candidatus* Izemoplasmatales (hereafter *Izemoplasma* and *Izemoplasmatales*). These orders are characterized by the lack of a peptidoglycan cell wall and are mostly host-associated, with small genomes and minimal metabolic capabilities [1–3]. There has been a long-standing controversy about the phylogenetic position of this lineage, in part due to inconsistent placement of fast evolving lineages such as mycoplasmas, that are subject to long branch attraction (LBA) artefacts [4]. Several comparative analyses that attempted to correct for LBA suggest a position within the phylum *Bacillota* (formerly known as the *Firmicutes*) [5–7]. According to the Genome Taxonomy Database (GTDB) [8], the *Mycoplasmatota* orders are placed within the class *Bacilli*, which are generally Gram-positive rods that are often capable of sporulation [3]. In the GTDB reference tree, the *Bacilli* order *Erysipelotrichales* is located amongst the *Mycoplasmatales* and related orders. The *Erysipelotrichales* includes cultured representatives with Gram-positive cell envelopes, with some species capable of sporulation [9, 10]. The picture is further complicated by the presence of uncultured lineages recently detected in environmental surveys – *RFN20*, *RF39* and *ML615J-28* [8] – some of which are inferred to have Gram-positive cell envelopes [11]. However, this set of orders form a monophyletic group and have faster evolutionary rates than other *Bacilli* (Fig. 1, average tip length).

Both cell envelope peptidoglycan and ability to form endospores contribute to the environmental resilience of free-living bacteria [12, 13]. During host association, however, these resistance mechanisms are less critical and, in some instances, actively deleterious. Peptidoglycan is recognized by the innate immune system, and bacteria have been observed to adopt transient L-form peptidoglycan-deficient phenotypes in response to immune activation [14, 15]. Moreover, the L-form transition has been found as a means of escaping infection by phages, which have an important role in modulating gut bacterial communities [16, 17]. Similarly, loss of sporulation in host-associated members of the *Bacillota*, such as the order *Lactobacillales,* is correlated with genome reduction and specialized metabolic capabilities leading to increased host adaptation and a decrease in host range [18].

To investigate the evolutionary history of peptidoglycan and sporulation loss within the class *Bacilli*, all high-quality genomes belonging to this class in GTDB release 06-RS202 were used to infer extant and ancestral phenotypes. The orders *RF39, RFN20* and *Ml615J-28* were all predicted to have mixtures of species with typical Gram-positive cell envelopes with and without the ability to sporulate, and peptidoglycan-deficient non-sporulators. Ancestrally, the transition to peptidoglycan-deficient and non-sporulating phenotypes within the class *Bacilli* appears to have occurred independently on multiple occasions often via intermediary phenotype ancestors. These phenotype losses are predicted to have occurred consecutively with independent adoption of increasingly host-associated lifestyles, with degree of phenotype loss correlated with closeness of the interaction.

## Methods

### Genome selection and annotation

Release 06-RS202 of the GTDB taxonomy was used for selection of genomes (Table S1, available in the online version of this article; [19]). Genome completeness and contamination were calculated using CheckM 1.1.3 using lineage_wf [20]. Sequences below 95 % completeness or above 5 % contamination were discarded. Remaining sequences were clustered at the strain level with dRep 2.6.2 at an average nucleotide identity (ANI) threshold of 0.99 and alignment fraction of 10%, and the best representative of each cluster was selected based on CheckM score [21]. The GTDB-Tk 1.7.1 classify workflow was used to confirm taxonomic assignment of the final set of sequences using GTDB release 202 as the reference [22]. Codetta 2.0 was used to predict codon tables [23]. Prodigal 2.6.3 was used to predict ORFs and generate protein sequence files using either table 4 or 11 based on the codon usage predictions [24]. Prodigal was run twice, once with the additional –c command to remove ORFs that did not have closed ends for calculation of average ORF length. Pseudogenes for any genome predicted to use codon table 11 were identified with Pseudofinder [25] using the eggNOG 5.0 database as a reference [26]. Genomes GCF_000195855.1 (*Mycobacterium leprae*), GCF_003697165.2 (*Escherichia coli*) and GCF_000012345.1 (*Pelagibacter ubique*) were used as additional references for pseudogene counts. Genomes were annotated using DRAM 1.1.1 (using CAZyDB 07-31-2019, dbCAN V8, Pfam 33.0 and Kofam 2020-02-02 databases) to identify KEGG orthology (KOs) [27]. Functional annotations were taken as a Boolean presence or absence per KO per sequence and then averaged at the family level with each family considered to have a gene if it was present in more than half of the sequences within it. These averages were also rounded to the nearest 0.1 fraction and converted to a score between 0 and 10 for the family.

### *De novo* phylogenetic tree generation

IQ-TREE 1.6.12 was used to create species trees from the GTDB-Tk user-sequences-only 120 marker-gene concatenated alignment from the classify workflow above (-m MFP -bb 10000 –safe) [28]. To reduce the number of tips in the tree for downstream application, each family was collapsed to a single tip. For each family, the node that contained all members of a given family with the fewest total descendants was considered the most recent common ancestor (MRCA). A new tip was placed at the MRCA node for each family, with an arbitrary branch length of 0.1 based on overall surrounding branch lengths, and all other tips were then pruned. The tree was then made ultrametric through the chronos function in the R package ape [29]. All branch lengths in the ultrametric tree were multiplied by 100 to increase total length for downstream calculations while retaining relative length.

### Environmental distribution

The biosample ‘isolation source’ field of all genomes from the class *Bacilli* with an associated NCBI biosample accession were obtained from the NCBI website. A set of common keywords (Table S2) was used to allocate most of these genomes into either ‘host-associated’ or ‘environmental’ categories.

### Ancestral genome content prediction

CAFE5 was run using the *de novo* tree and the family average number of KO copies to predict the gene content at the root of the tree and any nodes where those genes were lost, using Poisson distribution and five gamma-rate categories [30]. The consensus tree was then subdivided, taking both daughter nodes from the root node and generating new trees with the daughter nodes as the new root nodes. CAFE5 was run again on each of these subtrees to identify KOs predicted to be in the new root node that were not predicted to be in the original root node, indicating horizontal acquisition at that node. This prediction and subdivision process was repeated iteratively for every internal node. Any KOs that were predicted in a given root were then deleted from the completed analysis of any descendant subtrees. The predictions for all internal nodes were then pooled to give the complete reconstruction for each node (Table S3). In addition to this conventional use of gene copies, a second reconstruction was performed using a score of 0–10 for each KO in place of copy number, representing the family average Boolean presence of the gene in 0–100 % of representative genomes (Table S4). To benchmark the viability of this alternative score-based application, Zombi was used to generate a complete simulated evolutionary tree to benchmark the protocol at a similar scale, using the example Cyanobacteria dataset but with ‘origination’ rate of 0.1, ‘total lineages’ of 5000 and ‘initial genome size’ of 1500 (Fig. S1 [31]). The ‘extant’ rather than ‘complete’ tree was used to simulate extant sets with incomplete historical coverage due to extinctions. To simulate the family-average pooling and pruning in the experimental data set, all nodes exactly six speciations from the root were treated as ‘family’ ancestors of all their respective descendant nodes/tips.

### Gene trees and distance matrices

KOs involved in sporulation and peptidoglycan synthesis identified in at least one genome within each of the orders *Acholeplasmatales*, *RFN20, Ml615J-28, RF39* and *Erysipelotrichales* were used as candidates for the creation of gene trees and distance matrices. Alignments were prepared using MAFFT 7.481 and masked using trimAl 1.4.1 using default settings [32, 33]. IQ-TREE 1.6.12 was used to infer trees from the gene family multiple sequence alignments (-m MFP -bb 1000 –safe) [28]. EMBOSS was used to convert the multiple sequence alignments into distance matrices [34]. Distances were averaged across gene copies within each genome and then averaged across genomes at the family and order levels. These matrices were then averaged across all KOs by sporulation or peptidoglycan synthesis category.

### Additional genome selection and annotation

An additional data set containing all genomes in the GTDB 08 RS214 taxonomy was quality filtered and dereplicated at the species level [19]. Genome completeness and contamination were calculated using CheckM 1.1.3 using lineage_wf [20]. Sequences below 90 % completeness or above 5 % contamination were discarded. Species clusters with more than one genome passing these thresholds were dereplicated arbitrarily (Table S5). Genomes were annotated using HMMER 3.3.2 against a version of the Kofam 2022-01-12 database trimmed to only specific phenotypic genes of interest, using an E-value cutoff of 1e-23 to balance sensitivity and specificity of the analysis [35]. A subset of the genomes was also used for a complete annotation using DRAM 1.2.4 against the complete Kofam 2022-01-12 database [27]. The annotations for the trimmed and complete annotations were compared; only those gene families with less than an average annotation discrepancy of 5 % were retained for analysis. Annotations were taken as a Boolean presence or absence per genome and then averaged at the genus level, with the genus averages used to calculate the family average and so forth.

### Figures

R 4.0.3 and the packages pheatmap 1.0.12 and ggtree 3.6.2, along with their respective dependencies, were used to create figures [36–38].

## Results

### Genome selection and inference of reference topology

To predict the novel family phenotypes and ancestral states, only high-quality genomes (estimated completeness >95 %, contamination <5 %) in the class *Bacilli* (GTDB release 06-RS202) were used to reduce potential noise. However, this excluded many species and genera belonging to the as-yet-uncultured orders *RF39*, *RFN20* and *ML615J-28* (Table S6). As such, the novel orders were generally characterized in terms of broadly (order-level) conserved features and family-level variation rather than on a genus or species level. Families with single high-quality representatives were included in the analysis for purposes such as establishing broadly conserved features but were not themselves analysed in detail.

A *de novo* species maximum likelihood tree was inferred using the bac120 concatenated from the subset of high-quality *Bacilli* genomes. Given that this region of the tree is potentially affected by long branch attraction, we selected the best model (LG+F+R10) using ModelFinder [39] and inferred the tree using IQ-TREE, which is more robust to artefacts than FastTree used to infer the GTDB reference tree [8, 19, 22, 40]. This confirmed that the peptidoglycan-deficient lineages are interleaved with typical Gram-positive lineages (Figs 1 and S2), consistent with previous analyses [7, 11, 19]. Therefore, this tree was used to overlay genome statistics and for ancestral reconstruction, though nodes ancestral of *Bacillaceae_B* were not analysed in detail or referenced in the following sections.

### Genome statistics and isolation sources

Genome statistics including size, GC content, average family branch length, pseudogene content, hypothetical gene content, average gene length and tip branch length of families belonging to the three uncultured ingroup orders were compared against neighbouring cultured families for preliminary predictions of lifestyle and genome dynamics. The genome size and gene number of the three uncultured orders are similar to each other and, as with the *Izemoplasmatales,* are significantly larger than the *Mycoplasmatales* and significantly smaller than the *Erysipelotrichales* (Fig. 1 and Table S7). This suggests either some degree of host association, but not at the extreme obligate level of the *Mycoplasmatales*, or free-living bacteria with streamlined genomes for specialized lifestyles as seen in the *Izemoplasmatales*.

The family average branch length from the order common ancestor to the tips, indicative of evolutionary rate, displayed a strong inverse correlation (Pearson correlation coefficient −0.78) between tip length and genome size, indicating a trend between the fast-evolving lineages and genome reduction.

It has been observed that pseudogene content is reduced in highly streamlined free-living species, such as *Pelagibacter ubique* [41], and increased in species that have recently adopted an increased degree of host association [42], such as *Mycobacterium leprae* [43]. Consistent with this trend, the pseudogene annotation for the *Escherichia coli, Pelagibacter ubique* and *Mycobacterium leprae* reference genomes were 10.39, 1.92 and 36.32% respectively. Average pseudogene percentages among both the families of the uncultured orders and the broader class *Bacilli* were similar, in the range 3–10 %, suggesting similar levels of genomic streamlining between the uncultured and reference orders and that none of the uncultured orders have recently undergone a lifestyle transition.

The isolation sources of the genomes in the class *Bacilli* were used to calculate the relative distribution between host-associated and environmental niches (Fig. 1). For the ingroup references, most were primarily localized in host-associated niches. The exceptions were the *Izemoplasmatales* families, distributed across various water-based niches such as lakes and ocean sediment as expected [1, 2], and *Acholeplasmataceae*, split between plant host association and sediment- or soil-based environmental niches as expected [44, 45]. The uncultured families displayed a similar relative distribution with most being from gut habitats, consistent with the oxygen tolerance predictions above. This suggests a predominantly host-associated lifestyle for the uncultured families.

### Evolutionary history of consistent whole-genome reduction

To determine trends of gene family expansion and reduction, the total numbers of unique KOs for all extant families and reconstructed ancestral (interior) nodes were compared. We first estimated ancestral KO content and copy number averaged at the family level using CAFE. We then also substituted a score of 0–10, indicating gene presence in 0–100 % of a given family’s representative genomes, in place of copy number.

Both methods indicated a reduction of unique gene content in the ingroup set of families relative to their common ancestor. Indeed, almost all extant and internal nodes had lower unique gene content than their immediate parent node (Fig. S3). This pattern of gene loss is consistent with a trend of continued overall whole-genome reduction in these lineages.

### Restricted metabolic profile in novel orders

To predict overall metabolic and biosynthetic capabilities of the uncultured orders and ancestral reconstructions, the number of KOs in Brite category 09100 Metabolism was used to determine the relative total metabolic potential of the uncultured families and their parent orders. For the extant uncultured orders, the overall range of metabolic functions was lower than for the *Bacillales, Lactobacillales, Staphylococcales* and *Erysipelotrichales* references but higher than for *Mycoplasmatales*. The general pattern observed in the novel families was an absence of redundancy in pathways, the presence of salvaging rather than synthesis reactions, the presence of disconnected individual intermediate reactions and in some cases the absence of critical reactions (Fig. S3, Tables S3 and S4). The general lack of synthesis reactions for critical metabolites combined with the highly conserved presence of salvaging reactions suggests that the novel orders rely on their external environment to supply most cellular components either from a rich habitat such as the gut or a microbial host cell.

For the ancestral reconstructions, the overall trend of metabolic potential was one of consistent stepwise loss, with several instances of more substantial losses at nodes such as the common ancestor of the *Mycoplasmatales* (Fig. S3). Almost all internal nodes had a reduced number of metabolic genes compared to the immediate parent node. This is consistent with the prediction of consistent and independent genome reduction as seen for genome size above.

### Cell-wall-intermediate and peptidoglycan-deficient phenotype transitions

**Fig. 2.**
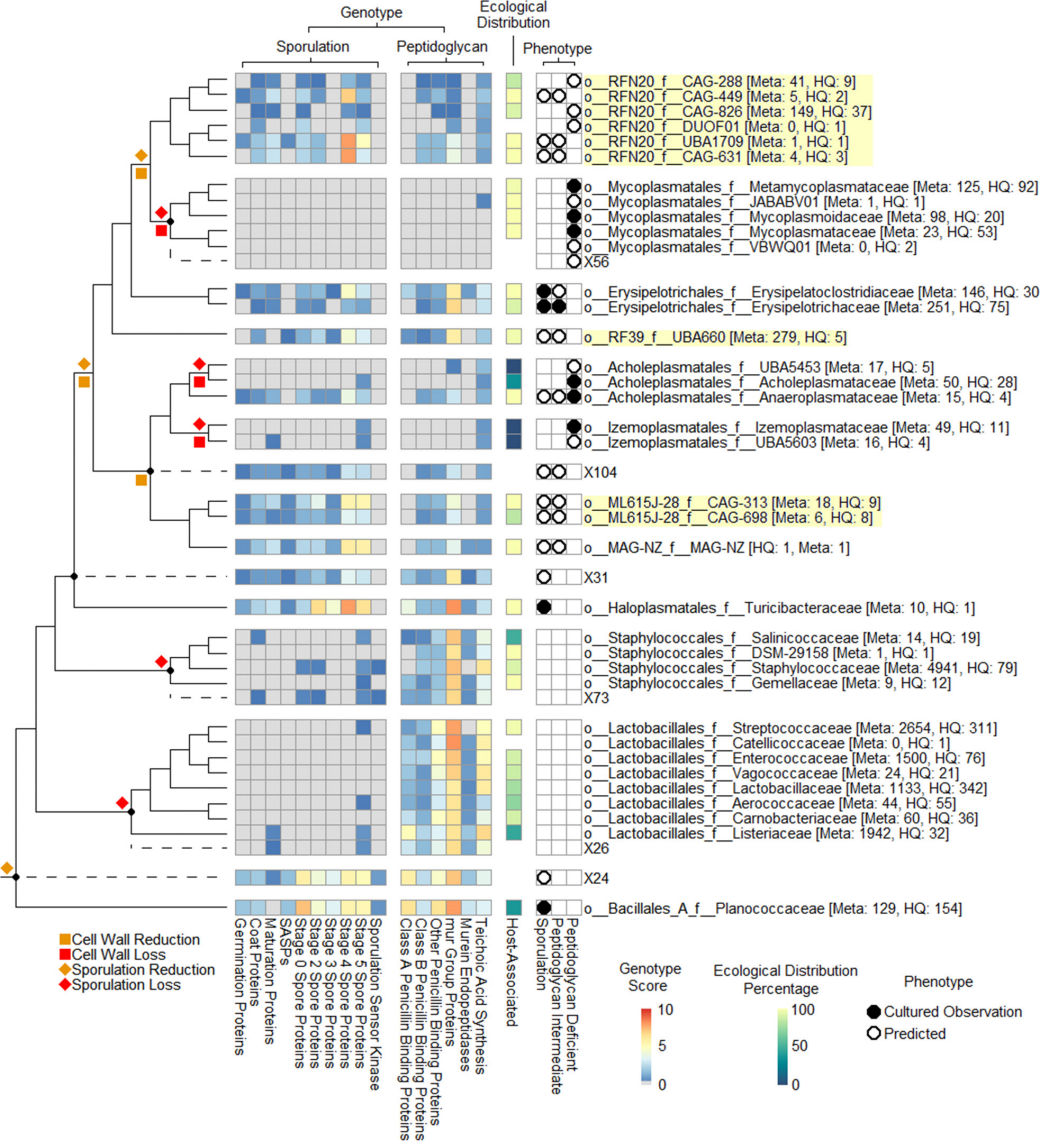
Cladogram of ingroup collapsed at the family level showing genome-based sporulation and peptidoglycan phenotype predictions, ecological distribution, and experimental observations. Phenotype predictions are based on individual KOs averaged by family average or the KOs inferred to be present in ancestral nodes (marked by dashed lines). Overall category values are averaged from all KO sub-averages in that category. Ecological distributionis are shown as a percentage of genomes sourced from host-associated niches. Phenotype is indicated by either filled circles (experimental observation in at least one species belonging to the family) or open circles (predicted at the family level based on overall gene content). Taxon names are prefixed with rank designations: o__, order; f__, family. The number of genomes used to infer the ecological distribution for each family (Meta) and the number of high-quality (HQ) genomes used for metabolic inference are shown in square brackets after the taxon names. Predicted evolutionary transitions associated with these phenotypes are shown at interior nodes to the left of the figure according to the inset key.

The presence or absence of a peptidoglycan in the novel families was predicted based on presence or absence of peptidoglycan synthesis and wall-component genes (Figs 2 and S4; Tables S3, S4 and S8). It was found that both families in the order *Erysipelotrichales* were lacking many of the expected genes involved in peptidoglycan synthesis in the *Bacillus* reference model, including the majority of penicillin-binding proteins (PBPs) and the *lyt[X]/cwl[X]* endopeptidases. In *Bacillus* models, these endopeptidases are vital for cell elongation, division and growth; redundancy between these genes means that single knockouts remain viable but multiple knockouts are typically fatal [46]. Likewise, PBPs have a range of functions involving cross-linking and integration of peptidoglycan precursors into the peptidoglycan [46]. *Erysipelotrichales* have been demonstrated as viable and typically exhibit rod morphology in culture, so these deficiencies would suggest a thin-wall phenotype rather than a classical Gram-positive wall for the order *Erysipelotrichales*, which is consistent with observations of rapid Gram-stain loss in some species [18, 47, 48]. The set of genes present in at least 50 % of genomes within both *Erysipelotrichales* families was taken as the baseline for minimum necessary genes for the presence of a thin-wall phenotype in the novel families.

The orders *RF39*, *ML615J-28* and *RFN20* family *CAG-631* were predicted to have the meso-diaminopimelic acid (*m-*DAP) type peptidoglycan synthesis pathway, though in all cases there were steps missing and some of the precursor synthesis reactions were absent. These missing reactions, without replacement via unidentified redundant genes, would indicate an inability to fully synthesize peptidoglycan *de novo* and introduce a requirement for muropeptides that would need to be scavenged from a host or the habitat more generally. For other peptidoglycan genes, *RF39*, *ML615J-28* and *RFN20* families *CAG-449* and *CAG-631* encode the majority of peptidoglycan genes found within *Erysipelotrichales*, while *RFN20* families *CAG-288* and *CAG-826* encode none of them in most species. An exception to this was the lack of the *srtA/B* sortases in the orders *RFN20* and *ML615J-28*. The *srtA/B* sortases are involved in attaching proteins to the peptidoglycan in the cell wall, with knockout *Staphylococcus* mutants shown to have reduced virulence and cellular adhesion [49]. Absence of these sortases in *RFN20* and *ML615J-28* probably indicates reduced surface protein expression. All novel families were predicted to possess a similar set of teichoic acid genes to the *Erysipelotrichales* references, though with sufficient losses that synthesis of wall teichoic acids and lipo-teichoic acids via the standard pathway is predicted to be absent.

Overall, we predict that essentially all species in *ML615J-28* family *CAG-313* and *RFN20* families *CAG-449* and *CAG-631*, as well as most species in *RF39* and *ML615J-28* family *CAG-698,* possess a peptidoglycan-containing cell wall similar to that of *Erysipelotrichales. RFN20* families *CAG-288* and *CAG-826* are predicted to be peptidoglycan-deficient. Interestingly, 40–60 % of genomes in the peptidoglycan-deficient reference family *Anaeroplasmataceae* contain peptidoglycan biosynthetic genes such as *mur* group genes, which may indicate the presence of some walled species in this family or vestigial genes from a more recent transition to a peptidoglycan-deficient phenotype.

The ancestral reconstructions were found to indicate multiple independent losses of peptidoglycan from the cell wall, possibly via intermediate phenotype transitions. The common ancestral node to *RFN20/RF39/ML615J-28* was predicted to have lost the majority of the PBPs and *lyt[X]/cwl[X]* endopeptidases, with both the nodes ancestral to the orders *Mycoplasmatales*/*RFN20* and *Izemoplasmatales*/*Acholeplasmatales* predicted to have lost racemases, ligases and flippases along with the various *mur* genes. These losses suggest that the ancestral species from which *Haloplasmatales* diverged either underwent some manner of peptidoglycan restructuring for which these genes were no longer required, or that their functions were supplanted by unidentified genes either acquired horizontally since the common ancestor with the *Bacillus* reference ancestor, or lost within the *Bacillus* reference ancestor. Secondary losses of sortases and assorted peptidoglycan and peptidoglycan precursor synthesis genes appear to have been lost at the nodes ancestral to *RFN20/Mycoplasmatales* and *Acholeplasmatales/Izemoplasmatales/ML615J-28*. Loss of these synthesis genes would introduce the requirement for availability of environmentally scavenged muropeptides for growth and the sortases would reduce cell surface proteins and cellular adhesion [49]. From there, independent and complete loss of the peptidoglycan appears to have occurred at the ancestor of the *Mycoplasmatales* and *Izemoplasmatales* as well as at or within the individual family level such as for *RFN20 CAG-449* and *Acholeplasmatales*. With some isolated exceptions, such as limited apparent horizontal transfer of genes *pbpA/2X* or *femA/B* within the orders *Staphylococcales* and *Lactobacillales*, the trend is of loss or stable conservation. Overall, these predictions suggest that the ancestor of *Haloplasmatales* retained a Gram-positive cell wall, but underwent a substantial remodelling of the peptidoglycan structure or process. The repeated and independent subsequent transition to a peptidoglycan-deficient state potentially indicates a reduced genomic stability for this atypical wall phenotype, being more susceptible to random loss, and/or that the atypical wall phenotype necessitated or facilitated growth in environments within which the loss of the peptidoglycan was not lethal.

### Endospore-intermediate and endospore-deficient phenotype transitions

As with predictions for a Gram-positive phenotype, the ability for the extant families to sporulate was predicted based on presence or absence of genes involved in sporulation (Fig. 2; Tables S3, S4 and S8). The presence of the *spo0A* gene has been observed as an absolute requirement for spore formation in the *Bacillota* [50]. However, the presence of individual sporulation marker genes such as *spo0A* alone does not necessarily indicate ability to sporulate, as species have been identified that encode many sporulation genes but have not been observed to form spores, within both sporulating and non-sporulating lineages [18, 51]. As with the peptidoglycan genes, it was found that all *Erysipelotrichales* lack many of the sporulation genes in the *Bacillus* reference model. Among the absent genes are essentially all small acid-soluble sporulation proteins, coat proteins, germination proteins, maturation proteins and the sporulation sensor kinase, along with substantial losses in sporulation regulators and the general stage genes. Knockout of these missing genes in the *Bacillus* model impair sporulation in a variety of ways, such as reducing resistance to heat, lowering rates of spore formation and allowing formation under a reduced set of stimuli [52–54]. Despite this, many members of the *Erysipelotrichales* such as *Erysipeloclostridium ramosum* have been experimentally demonstrated to produce spores, and therefore the set of genes present in the majority of genomes of both families was taken as a baseline for the minimum complement of genes required for sporulation [18].

The order *ML615J-28* has a reduced presence or complete absence of *spoIID, spoIVFB*, *spoIIE*, *yabG, yqfD* and *spoVB*, which are present in sporulating *Erysipelotrichales*. Of these, *spoIID* is the only gene considered critical for sporulation as it is involved in degradation of the septal peptidoglyan [55]. However, a reduced set of ~25 sporulation genes persist in many members of the *ML615J-28*, ~50 % of family *CAG-698* representatives and ~90 % of family *CAG-313* representatives, suggesting that sporulation may still be possible in some limited fashion. Based on knockout studies, we predict that if spores are produced, they will have some or all of the following deficits: an altered coat (*yabG* [56]), reduced heat tolerance (*spoVB* [57]), lack of the classical σ^K^ activation mechanism (*spoIVFB* [58, 59]) and substantially reduced production (*spoIIE* and *yqfD* [55, 60]).

For the order *RFN20*, the families *CAG-288* and *CAG-826* are predicted to be unable to sporulate due to the lack of *spo0A*. *CAG-631* and *CAG-449* encode *spo0A* and many of the core set of sporulation genes present in the *Erysipelotrichales* so are predicted to form spores. However, most *CAG-631* and *CAG-449* lack *gerQ* and *spoIID,* which would reduce the number of environmental stimuli able to induce spore formation (*gerQ* [61]). Most *CAG-449* also lack *cotJB*, *gpr*, *spoIIE* and *spoVAD* but have an additional non-essential germination protein *yaaH* [62], and many of the sporulation genes that were present were not present in all species. As such, most *CAG-449* are predicted to be capable of forming spores, though with reduced spore stability (*spoVAD* [63, 64]), with reduced heat tolerance (*spoVAD*) and at substantially reduced rate (*gpr* and *spoIIE* [60, 65]) compared to *CAG-631*.

Compared to the *Erysipelotrichales* reference, *RF39* had a reduced presence or complete absence of *gerQ*, *spmB*, *spoIIAB*, *spoIIE*, *spoVB* and *spoVD*, though with the additional presence of *spoIIGA, spoIIM, spoIIP, spoIIQ, spoIIAH* and *spoVG*. These additional genes are critical to the activation and/or localization of σ^E^ and σ^G^ along with septal peptidoglycan degradation [66]. As such *RF39* is predicted to be capable of forming spores, though with a markedly different phenotype and regulation than *Erysipelotrichales*.

Although not the main focus of the present study, we noted that a majority of genomes belonging to the non-spore-forming family *Anaeroplasmataceae* contain sporulation genes (including *spo0A*) that, similar to the unexpected prediction of cell walls in some as-yet uncultured members of this family, may indicate that some *Anaeroplasmataceae* are spore-forming.

Ancestrally, there was a substantial reduction in the number of predicted sporulation genes over the two ancestral nodes from which the family *Jeotgalibacillaceae* and order *Lactobacillales* diverged, resulting in the loss of all sporulation-related small acid-soluble proteins, almost all germination proteins, approximately half the regulators and many of the coat proteins (Fig. 2). The *spo[n]* sporulation stage genes along with the remaining regulators and coat proteins were largely retained in the subsequent ancestral nodes, but with additional, less extreme losses of extra genes across the categories. Complete loss of spore genes, at the whole-family or higher level, appears to have then occurred multiple independent times, at the ancestors of the following orders: *Mycoplasmatales*, *Lactobacillales*, *Staphylococcales*, *Izemoplasmatales*, and the ancestral nodes for families *Acholeplasmataceae* and *UBA5453* in the order *Acholeplasmatales,* and at one or more points within the order *RFN20*. There do not appear to be any internal nodes at which horizontal gene transfer of sporulation-related genes occurred, nor does there appear to be any instances of a gain of sporulation phenotype. A possible explanation for this evolutionary trend is that the transition to the restricted set of sporulation genes, such as the loss of regulators and coat proteins, reduced the range of conditions under which sporulation could occur and be effective, leading to further independent losses by species in niches for which the impaired phenotype was not useful.

### Distinguishing between multiple losses and multiple gains

To determine whether the predicted interleaving of peptidoglycan-deficient and sporulating lineages was the result of multiple independent losses and/or multiple independent reacquisitions, individual gene trees and aggregate distance matrices were created from alignments of genes involved in sporulation and peptidoglycan that were conserved among all the ingroup lineages. These gene trees and aggregate distance matrices (Fig. S5, File S1) reflected species evolution, i.e. primarily vertical transmission in the orders *Acholeplasmatales, ML615J-28, RF39, RFN20, Erysipelotrichales* and *Haloplasmatales*. This is consistent with a common ancestor and multiple independent loss events, rather than loss followed by subsequent reacquisition from non-ingroup lineages. However, the relative position of the ingroup to the other orders in the class *Bacilli* was less consistent, which could potentially indicate a more extensive loss with a partial reacquisition prior to the common ancestral node of the orders of interest.

### Observed evolutionary trends are ongoing and mirrored in surrounding phyla

**Fig. 3.**
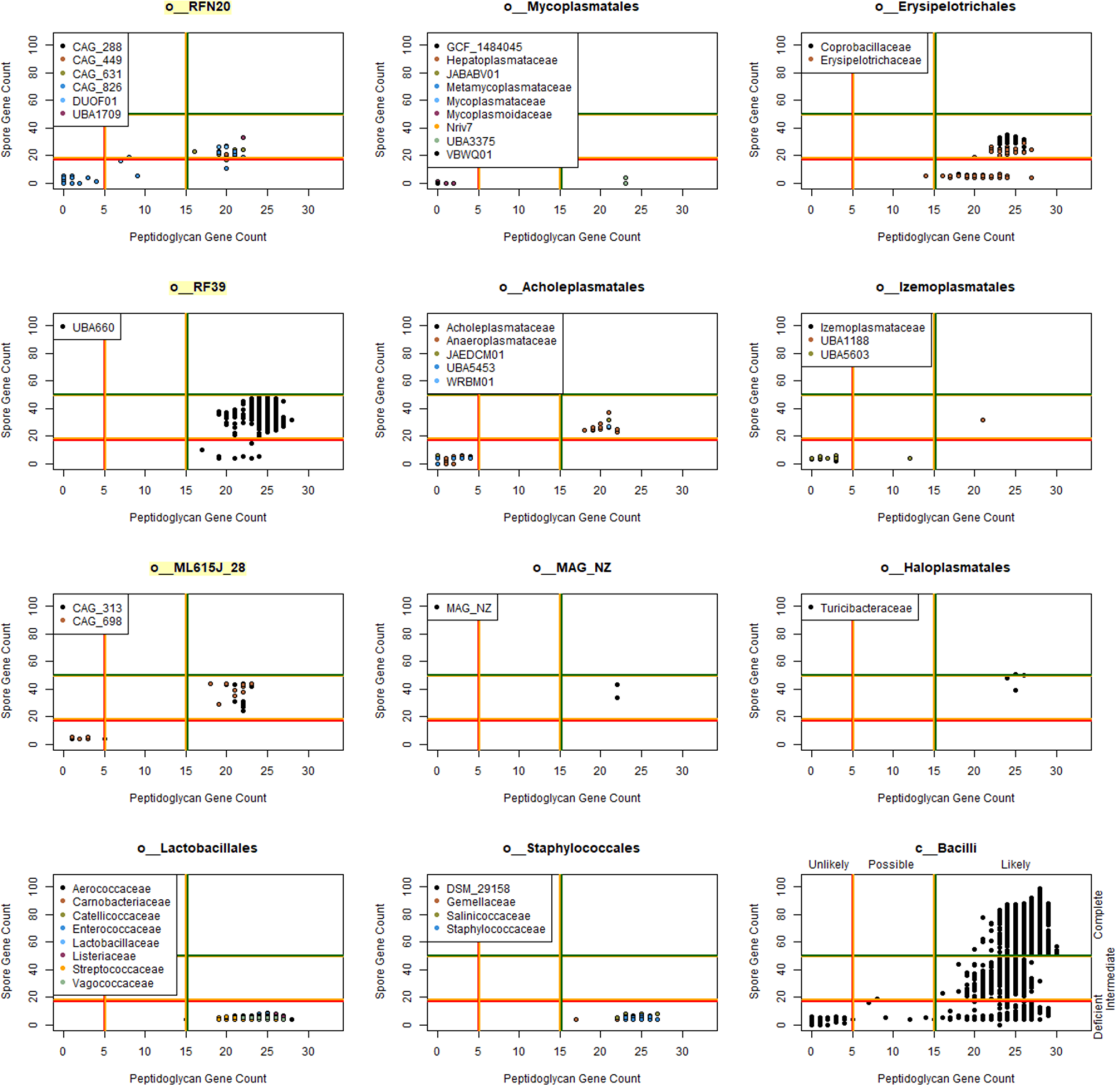
Scatterplots of spore gene count against peptidoglycan gene count for ingroup lineages with the class *Bacilli* as a reference (bottom right). Vertical and horizontal lines indicate approximate phenotype transitions between full, intermediate and lost (indicated in the bottom right panel).

**Fig. 4.**
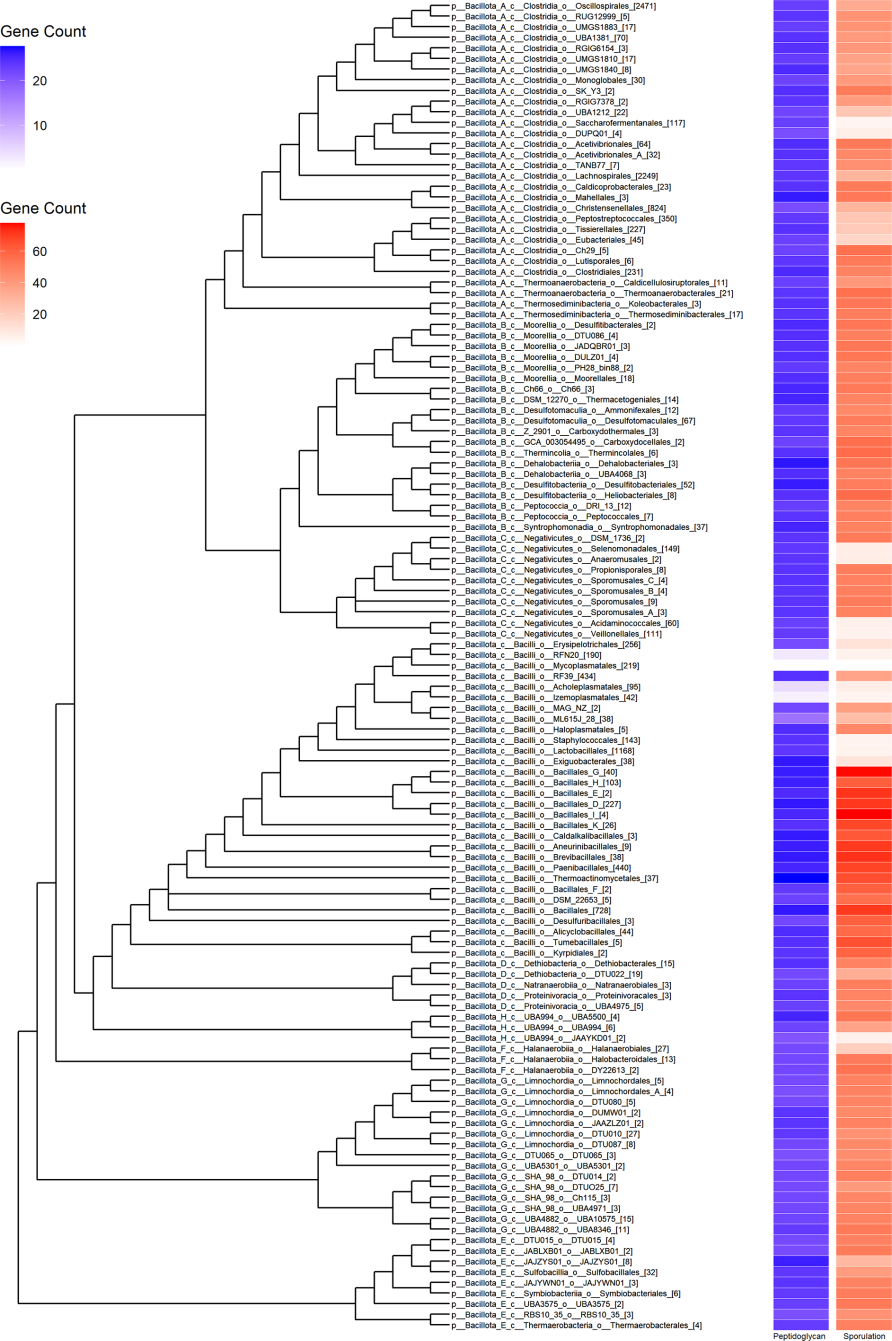
Cladogram of the phylum *Bacillota*. The number of high-quality genomes within each order is shown in square brackets after the names in the cladogram. Sporulation and peptidoglycan gene counts are shown to the right of the cladogram, displaying loss of these genotypes in the broader context of the ingroup. Gene counts were averaged across all KOs by subcategory and by order average.

Following these evolutionary observations at the family level and the release of the greatly expanded set of genomes within GTDB 08-RS214, an additional analysis was conducted at the individual genome level within the class *Bacilli* and at the order level within the surrounding classes and phyla. This expanded data set revealed numerous additional instances of phenotype loss at the intra-family or intra-genus level (Figs 3 and S6). Further, the expanded data set revealed additional instances of walled and/or sporulating phenotypes within the *‘plasmatales’* reference orders, such as *Mycoplasmatales* family *UBA3375*. Across those families and/or orders containing genomes across multiple cell-wall and/or spore-forming states, there were two observed trends. In most cases, loss of sporulation genes was concurrent with a loss of cell-wall genes, possibly indicative of a general relaxation in selection for environmental persistence mechanisms as these lineages transition to host-associated lifestyles (Fig. 3). However, some lineages including *Lactobacillales* and *Staphylococcales* demonstrated a complete loss of sporulation genes with essentially no reduction in cell-wall genes, suggesting that an intact cell envelope is important for the survival of these free-living lineages. There were no observed instances of large-scale loss of cell-wall genes without a corresponding loss of sporulation genes, as expected given the peptidoglycan composition of the endospore cortex. Annotation across all *Bacillota*_[X] phyla demonstrated a similar pattern of interleaving between spore-forming and non-spore -lineages at the order level as seen in the class *Bacilli* (Fig. 4). These results suggest that the evolutionary trends in cell-wall and sporulation phenotype seen within the class *Bacilli* ancestral state reconstructions is both an ongoing process within *Bacilli* and the same trend is apparent for loss of sporulation genes in neighbouring classes.

## Discussion

Members of the class *Bacilli* encompass a range of Gram-positive sporulating, Gram-positive non-sporulating and peptidoglycan-deficient non-sporulating phenotypes within a tree topology that precludes single ancestral loss events of these key phenotypes. The formation of Gram-both positive peptidoglycan and endospores within the *Bacillus subtilis* reference model are complicated processes involving numerous and sometimes overlapping genes [46, 67]. However, comparisons against the sporulation machinery in members of the class *Clostridia* demonstrate the potential for diversity of the sporulation genes and phenotype across various lineages [50]. Similarly, though under a Gram-negative model, certain *Rickettsia* species have been demonstrated to have an intermediate state between a classical Gram-negative and peptidoglycan-deficient phenotype due to the loss of Class A PBPs [68], which is consistent with the observed absence of Class A PBPs in the *Erysipeloltrichales*.

Annotations of the extant *Bacilli* genomes in GTDB release 06-RS202 here revealed that characterized Gram-positive sporulating species within the order *Erysipelotrichales*, such as *Erysipeloclostridium ramosum* and *Erysipelatoclostridium spiroforme*, are missing numerous genes involved in both sporulation and peptidoglycan synthesis (Tables S3 and S4). Such losses, including some genes considered to be essential in the *Bacillus* model, would suggest more intermediate thin-wall and fragile-spore phenotypes. This hypothesis is supported in part by experimental observations of rapidly destaining Gram-positive cells and variable reports of spore formation in some members of the *Erysipelotrichales* [47, 69]. However, the nature of predictive genomics necessitates complete phenotypic models for accuracy and these predictions are predicated upon incomplete observations. To that end, further studies to investigate the specific composition and phenotypic properties of the cell envelope and endospores of the order *Erysipelotrichales* would be illuminating. Comparative predictions of uncultured novel lineages against *Erysipelotrichales* suggest that within the orders *Acholeplasmatales, RFN20* and *ML615J-28* there are a mix of thin-walled and peptidoglycan-deficient species, along with a mix of fragile-spore and non-sporulating species. Likewise, the order *RF39* is predicted to possess a thin wall with a mix of sporulating and non-sporulating species.

The natural evolutionary trend in bacterial genomes is reduction in absence of selective pressure [70]. Consistent with this, it has been observed in directed evolution experiments on *Bacillus* that sporulation is lost over a prolonged period of relaxed selection pressure [71]. Adoption of a host-associated lifestyle alleviates many environmental stressors. Sporulating bacterial lineages can maintain a vegetative phenotype within gut microbiomes, producing endospores for environmental persistence between hosts [72]. The necessity for endospores to transmit between hosts is correlated with the degree of environmental horizontal versus maternal vertical microbiome transmission, with many gut bacteria found to be former spore-formers [18]. A transition to a tighter host association further reduces the necessity for environmental resistance mechanisms, such as seen in the loss of the peptidoglycan in the osmotically protected niche occupied by endosymbiotic bacteria [73]. Using ancestral state reconstructions of the common ancestors of order- and family-level lineages in the class *Bacilli,* we show a trend of whole-genome reduction and a reduction in metabolic capabilities consistent with transitioning to increasingly host-associated lifestyles. For sporulation and the peptidoglycan specifically, there appear to have been multiple independent loss events at both the ancestral and the individual family level or below. The complete loss of these phenotypes appears to have in some cases been via intermediate phenotype transitions, such as thin-wall and fragile-spore phenotypes (Fig 3). Our observations suggest a long and complex history of independently increasing degrees of host association, either driven by or resulting in decreasing metabolic capabilities and reduced environmental resistance within the class *Bacilli*.

## Supplementary Data

Supplementary material 1
